# Supplementary material for: Anomalous Interfacial Electron-Transfer Kinetics in Twisted Trilayer Graphene Caused by Layer-Specific Localization
Source: ACS Cent Sci. 2023 May 15;9(6):1119–28. doi: 10.1021/acscentsci.3c00326 (PMC10311658; doi:10.1021/acscentsci.3c00326)
Supplement: Supplementary file 1 — oc3c00326_si_001.pdf [file oc3c00326_si_001.pdf]

Supporting Information for

Anomalous interfacial electron transfer kinetics in twisted trilayer graphene caused by layer-specific localization

Kaidi Zhang[1], Yun Yu[1,†], Stephen Carr[2], Mohammad Babar[3], Ziyan Zhu[4], Bryan Kim[1], Catherine Groschner[1], Nikta Khaloo[1], Takashi Taniguchi[5], Kenji Watanabe[6], Venkatasubramanian Viswanathan[3], and D. Kwabena Bediako*[1,7]

[1] *Department of Chemistry, University of California, Berkeley, CA 94720, USA*
[2] *Brown Theoretical Physics Center, Brown University, Providence, RI, USA*
[3] *Department of Mechanical Engineering, Carnegie Mellon University, Pittsburgh, PA, USA*
[4] *SLAC National Accelerator Laboratory, Stanford, CA, USA*
[5] *International Center for Materials Nanoarchitectonics, National Institute for Materials Science, Tsukuba, Japan*
[6] *Research Center for Functional Materials, National Institute for Materials Science, Tsukuba, Japan*
[7] *Chemical Sciences Division, Lawrence Berkeley National Laboratory, Berkeley, CA 94720, USA*
[†] *Current affiliation: Department of Chemistry and Biochemistry, George Mason University, Fairfax, VA, USA*
[*] *Correspondence to: bediako@berkeley.edu*

Contents

1 Supplementary Text S2

2 Supplementary Figures S13

3 Supplementary Tables S32

1 Supplementary Text

Raman mapping of ABA and ABC graphene

The Raman maps for ABA/ABC trilayer graphene were obtained from the experimental Raman map data by fitting Lorentzian functions on spectra collected at each grid point. A Lorentzian function is defined as the following:

$$L(x) = \frac{A}{\pi}\left(\frac{\frac{\Gamma}{2}}{(x - x_0)^2 + \frac{\Gamma}{2}}\right) \tag{1}$$

where A is the amplitude, x_0 is the peak center, and $\frac{\Gamma}{2}$ is the half-width at half-maximum (hwhm). The full width at half maxima (fwhm) was obtained using fwhm = 2 × hwhm for each grid point's fitted Lorentzian function. The grid points were then colored using a diverging colormap, with the color red corresponding to larger fwhm, blue for smaller fwhm, and white for intermediate fwhm (see SI Figs. 13,14).

Extracting areal fraction from STM and dark field images

STM images (see Fig. 4, SI Fig. 10) and dark field TEM images (see SI Fig. 4) were used to obtain information on the distribution of different stacking orders in twisted trilayer graphene. For STM images, the AAB/AAA bright spot size was obtained from measuring height profile line scans across STM data (see SI Fig. 5). The height profiles were smoothened with a moving average window of 5 nm, then the peak locations were extracted with a peak-finding function. Gaussian functions were then fitted to each peak, and the full width at 10% maximums was approximated to be the AAB/AAA bright spot radius. For dark field images, the different stacking domains were fitted based on intensity differences.

Finite element simulation

The finite-element simulation for the steady-state cyclic voltammograms was conducted on COMSOL Multiphysics (version 5.6). We utilized a similar program as in reference 1. The geometry of the pipette was built in a 2-D axisymmetric model as shown in SI Fig. 15. The radii of the droplet were assumed to be the same as the aperture of the pipette based on previous studies on twisted bilayer graphene[1]. The modules "transport of dilute species" and "electrostatics" were used in conjunction to simulate the mass transport of redox-active molecules in the electrochemical cell defined by the nanopipette and the meniscus. The steady-state mass transport of redox species was simulated by solving the Nernst–Planck equation:

$$D_i(\frac{\partial^2 c_i}{\partial r^2} + \frac{1}{r}\frac{\partial c_i}{\partial r} + \frac{\partial^2 c_i}{\partial z^2}) = -\frac{z_i F c_i D_i}{RT}(\frac{\partial^2 \phi}{\partial r^2} + \frac{1}{r}\frac{\partial \phi}{\partial r} + \frac{\partial^2 \phi}{\partial z^2});\ 0 < r < r_s, 0 < z < l \tag{2}$$

where r and z are the coordinates in directions parallel and normal to the sample surface, respectively. r_s and l are the width and the height of the simulation space, respectively. $l = 30m$ is set to ensure that the simulation space is much larger than the aperture size. The meniscus formed between the nanopipette and the sample surface was presented as a cylinder with a height of h. The contact (electroactive) radius, a_s, was set equivalent to the nanopipette radius, a, consistent with previous reports[1–3]. c_i, z_i, and D_i are the concentration, charge number, and diffusion coefficient of either the oxidized form (c_O) or the reduced form (c_R). ϕ is the electric potential in solution, solved by the Poisson equation:

$$\frac{\partial^2 \phi}{\partial r^2} + \frac{1}{r}\frac{\partial \phi}{\partial r} + \frac{\partial^2 \phi}{\partial z^2} = -\frac{\Sigma_i z_i F c_i}{\varepsilon \varepsilon_0};\ 0 < r < r_s, 0 < z < l \tag{3}$$

where $\varepsilon = 80$ is the dielectric constant of the solvent water and ε_0 is the vacuum permittivity. The c_i and z_i in equation S-7 include the ions of the supporting electrolyte (0.1 M KCl) in addition to the redox active species c_O and c_R. The rate of heterogeneous electron-transfer reaction is governed by the Butler–Volmer equations:

$$k_{red} = k^0 e^{-\alpha \frac{F}{RT}(V_{app} - E^0)} \tag{4}$$

$$k_{ox} = k^0 e^{(1-\alpha) \frac{F}{RT}(V_{app} - E^0)} \tag{5}$$

where k^0 is the standard rate constant, α is the transfer coefficient, F is the Faraday constant, E^0 is the standard potential, and V_{app} is the applied electrochemical potential. For the simulation of $Ru(NH_3)_6^{3+/2+}$, only the oxidized form (c_O) is initially present in the solution. For $Co(phen)_3^{3+/2+}$, equal concentration of the oxidized form (c_O) and reduced form (c_R) were present. Flux was considered to be zero apart from the contact surface. The general boundary conditions are shown below:

$$c_O = c_O^*, c_R = c_R^*;\ 0 < r \leq r_s, z = l; (bulk) \tag{6}$$

$$\frac{\partial c_i}{\partial n} = 0;\ 0 < z \leq h, r = a_s;\ h < z < l, r = a + (z - h)\tan(\theta_p); (no\ flux) \tag{7}$$

$$J_O = -J_R = k_{red} c_O - k_{ox} c_R; 0 < r \leq a_s,\ z = 0;\ (sample\ surface) \tag{8}$$

where J_O and J_R are the inward flux of the oxidized and reduced form, respectively. c_O^* and c_R^* are the bulk concentrations, $\frac{\partial c_i}{\partial n}$ is the normal derivative of concentration. The potential drop across the Helmholtz layer was implemented by defining the surface charge density, σ, at the sample surface:

$$\sigma = (V_{dl} - \phi)\varepsilon_H \varepsilon_0 / d_H;\ 0 < r \leq a_s,\ z = 0; \tag{9}$$

where $\varepsilon_H = 6$ and $d_H = 0.5$ nm are the dielectric constants and the thickness of the Helmholtz layer yielding $C_{dl} = 10$ µF/cm^2. V_{dl} is the double-layer potential relative to the charge neutrality point. The steady-state current was evaluated by integrating the total flux of the reactants (J_O) normal to the sample surface:

$$i = 2\pi F \int_0^{a_s} J_O r dr \tag{10}$$

For studies of the $Ru(NH_3)_6^{3+/2+}$ couple, D_O and D_R were set to 8.43×10^{-6} cm^2/s and 1.19×10^{-5} cm^2/s[4]. $\alpha = 0.5$ was used for all simulations consistent with previous studies on graphene thin films[1,5]. We also confirmed α by comparing with simulated voltammograms of different α values (see SI Fig. 8) and it is close to 0.5 across all twist angles we investigated. E^0 was determined from electrochemically reversible voltammograms obtained on a gold or platinum electrode right before the experiments on graphene. For studies of the $Co(phen)_3^{3+/2+}$, $D_O = D_R = 3.7 \times 10^{-6}$ cm^2/s were used for simulation. The pipette taper angle, θ_P, was consistent with pipettes fabricated with the same program reported in a previous study (see SI Fig. 11)[1].

To extract k^0 from an experimental curve, we generated a series of simulated voltammograms with varying k^0 values and evaluated the residuals of each relative to the experimental curve (based on a sigmoidal fit of the voltammogram), to obtain the coefficient of determination (R-squared, R^2) of each simulated curve. The best fit is determined from the highest R^2 value which is equivalent to the one with the lowest residuals. This procedure is demonstrated in SI Fig. 8 where the R^2 values versus different simulated rates are plotted in the bottom right insets of the representative voltammograms.

Marcus-Hush-Chidsey calculation

Theoretical electrochemical rate constants shown in SI Fig. 7 were calculated using the Marcus–Hush–Chidsey formalism:

$$k_{\mathrm{ox}} \propto \int D\left(\epsilon - eV_{\mathrm{q}}\right) \exp\left[-\frac{(\lambda - E^0 + \epsilon)^2}{4\lambda k_{\mathrm{B}}T}\right] \frac{\exp\left(\frac{\epsilon}{k_{\mathrm{B}}T}\right)}{1+\exp\left(\frac{\epsilon}{k_{\mathrm{B}}T}\right)} \mathrm{d}\epsilon \tag{11}$$

$$k_{\mathrm{red}} \propto \int D\left(\epsilon - eV_{\mathrm{q}}\right) \exp\left[-\frac{(\lambda + E^0 - \epsilon)^2}{4\lambda k_{\mathrm{B}}T}\right] \frac{\mathrm{d}\epsilon}{1+\exp\left(\frac{\epsilon}{k_{\mathrm{B}}T}\right)} \tag{12}$$

where $\lambda = 0.82$ eV is the reorganization energy for the $Ru(NH_3)_6^{3+/2+}$ redox couple[6], E_0 is the energy level of the formal potential, k_B is the Boltzmann constant and $D(\epsilon)$ is the DOS. We assume that the DOS is aligned with the molecular energy levels such that $\epsilon_F = 0$. The charge neutrality point of trilayer graphene relative to E^0 is assumed to be the same as bilayer graphene previously reported[1].

Area fraction analysis

The area fraction (AF) of a rigid moiré unit cell was calculated based on a simple geometric model of a half hexagonal unit cell shown in SI Fig. 16. We assumed the high-energy stacking domains (AAB, AAA, or ABB) are circles with diameter d. In a rigid moiré, we took $d = 0.5\lambda_m$ and the other stacking domains to occupy equal radians[7]. This leads to the rigid moiré area fraction reported in Fig. 4G and Supplementary Table 3. In a relaxed moiré unit cell, the diameter of the high energy stacking domains shrinks for small twist angles. Based on theoretical calculations, these stacking domains can be assumed to have a constant diameter at small twist angles (< 1 °)[8]. The saddle points (SP) can be considered to have a constant width as shown in Fig. 4. Therefore, the area fractions of high-energy stacking domains and saddle points can be calculated with the following equations based on geometric analysis:

$$AF\ of\ AAB/AAA = \frac{\pi d^2}{2\sqrt{3}a^2}\ sin^2(\frac{\theta_m}{2}) \tag{13}$$

$$AF\ of\ SP = \frac{4\sqrt{3}\delta}{a}\ sin(\frac{\theta_m}{2}) - \frac{F(d,\delta)}{a^2}\ sin^2(\frac{\theta_m}{2}) \tag{14}$$

where d is the width of the AAA or AAB stacking domain, $a = 0.246$ nm is the lattice constant for graphene, δ is the width of the SP, θ_m is the moiré twist angle, and $F(d,\delta)$ is a function of both d and δ. At small twist angles (< 1 °) when d and δ can be considered relatively unvaried, the AF of AAA, AAB, and SP can be fitted to a sine square function with respect to $theta_m$. We obtained the following equation based on STM and dark field images:

$$AF\ of\ AAB = 2035.33\ sin^2(\frac{\theta_m}{2}) \tag{15}$$

$$AF\ of\ AAA = 2223.19\ sin^2(\frac{\theta_m}{2}) \tag{16}$$

$$AF\ of\ SP = 107.09\ sin(\frac{\theta_m}{2}) - 7932.33\ sin^2(\frac{\theta_m}{2}) \tag{17}$$

The fitted equations were used to determine the area fraction reported in Supplementary Table 2. Area fractions of twist angle larger than 1° were assumed to be the same as those of a rigid moiré. We also derived the average diameter of AAB and AAA stacking domains based on the prefactor of the sin square equation. They were $d_{AAB} = 11.65$ nm and $d_{AAA} = 12.18$ nm. The mean local twist angle in AAA/AAB was determined by averaging the calculated twist angles on the pixels within the radii from the center of AAA/AAB domains (see SI Fig. 17).

Calculation of relaxation and local twist angle of TTL

To obtain the relaxation-redistributed local twist angle for A-t-A and M-t-B, we employed a continuum model to account for in-plane distortions due to relaxation and the generalized stacking fault energy (GSFE) to account for the interlayer coupling[9]. The relaxation of the layer i, $u_i(b)$, is defined in terms of the local configuration or the relative local stacking, b, and we obtain $u_i(b)$'s by minimizing the total energy[10]. The total energy has two contributions. The first is the intralayer energy of the i-th layer, which is calculated based on linear elasticity theory,

$$E_{\text{intra}}[u_i(b)] = \int_\Gamma db\, \frac{1}{2}\mathcal{E}({}_b u_i(u_i))\, C_i\, \mathcal{E}({}_b u_i(b))$$

$$= \int_\Gamma db\, \frac{1}{2}\left[G_i(\partial_x u_{i,x} + \partial_y u_{i,y})^2 + K_i((\partial_x u_{i,x} - \partial_y u_{i,y})^2 + (\partial_x u_{i,y} + \partial_y u_{i,x})^2)\right], \tag{18}$$

where Γ is the union of all configurations, $\mathcal{E}(u_i)$ is the strain tensor, C_i is the linear elasticity tensor of the i-th layer (which is identical for all i's in this case), with G_i and K_i being the shear and bulk moduli of the corresponding layer. Note that for M-t-B, we consider the AB bilayer graphene as an effective monolayer because there is no relative twist angle. For monolayer graphene, we take $G_i = 47352\,\text{meV/cell}$, $K_j = 69518\,\text{meV/cell}$ and the graphene unit cell size is $5.3128\,\text{Å}^2$[8]. For AB bilayer graphene, the shear and bulk moduli are twice as those of monolayer graphene. The gradient in Eq. (18) is with respect to the real space position $\vec{r}$, and following linear transformation maps the relaxation from the local configuration to the real space positions r:

$$b = (E_1^{-1}E_2 - 1)r, \tag{19}$$

where E_1 and E_2 are the unit cell vectors of the first and the second layers respectively, and $E_2 = \mathcal{R}^{-1}(\theta)$, where $\mathcal{R}(\theta)$ is the clockwise rotation matrix by θ.

The second energy contribution is the interlayer energy, which is described by the GSFE, $V_{\text{GSFE}}(b)$[8,9,11,12]. The GSFE is the relative energy difference between different stacking configu-

rations of the two adjacent layers, which is obtained by applying a 9×9 grid sampling of rigid shifts to layer 1 in the unit cell with respect to layer 2 and extract the relaxed ground state energy at each shift from density functional theory (DFT) with the Vienna Ab initio Simulation Package (VASP)[13–15]. We used the semi-local meta-GGA functional SCAN+rVV10[16] for its good performance in van der Waals materials and low computational cost. The GSFE of graphene at a given configuration $b = \begin{pmatrix} v & w \end{pmatrix}^T$ can then be expressed as follows,

$$\begin{aligned} V_{\text{GSFE}}(v, w) = c_0 &+ c_1(\cos v + \cos w + \cos(v + w)) \\ &+ c_2(\cos(v + 2w) + \cos(v - w) + \cos(2v + w)) \\ &+ c_3(\cos 2v + \cos 2w + \cos(2v + 2w)) \\ &+ c_4(\sin v + \sin w - \sin(v + w) \\ &+ c_5(\sin(2v + 2w) - \sin(2v) - \sin(2w)) \end{aligned} \tag{20}$$

where c_i's are coefficients found by fitting the ground state energy at each shift, and their values can be found in Table 1. Note that c_4 and c_5 for the graphene/graphene interface are due to the inversion symmetry between AB and BA stackings. In terms of the V_{GSFE}, the interlayer energy E_{inter},

$$E_{\text{inter}} = \sum_{i=1}^{L-1} \int_{\Gamma} db\, V_{\text{GSFE}}(b + u_i(b) - u_{i+1}(b)), \tag{21}$$

where L is the total number of effective layers and $L = 3$ for A-t-A graphene and $L = 2$ for M-t-B. The total energy is the sum of the interlayer and the intralayer energies:

$$E_{\text{tot}}(u(r)) = \sum_{i=1}^{L} E_{\text{intra}}(u_i(b)) + E_{\text{inter}}. \tag{22}$$

We then obtained the relaxation displacement in configuration space by minimizing the total energy with respect to $u_i(b)$.

	c_0	c_1	c_2	c_3	c_4	c_5
Monolayer/monolayer	6.832	4.359	-0.374	-0.095	0	0
Monolayer/bilayer	7.609	4.359	-0.126	-0.0122	0.0798	0.217

Table 1: GSFE coefficients between monolayer/monolayer graphene (top) and monolayer/AB bilayer graphene (bottom). All units are meV/unit cells.

After converting relaxation displacement vector in real space, $\vec{U}(\vec{r})$, via Eq. (19), we calculated the local twist angle between layers i and j as redistributed by relaxation as follows,

$$\theta_{ij,\text{local}}(\vec{r}) = \left|\theta_{ij} + \sin^{-1}\left(\frac{\vec{\nabla} \times \vec{U}^{(j)}(\vec{r})}{2}\right) - \sin^{-1}\left(\frac{\vec{\nabla} \times \vec{U}^{(i)}(\vec{r})}{2}\right)\right|. \tag{23}$$

The local twist angle distribution of several A-t-A and M-t-B were shown in SI Figs. 3,17.

2 Supplementary Figures

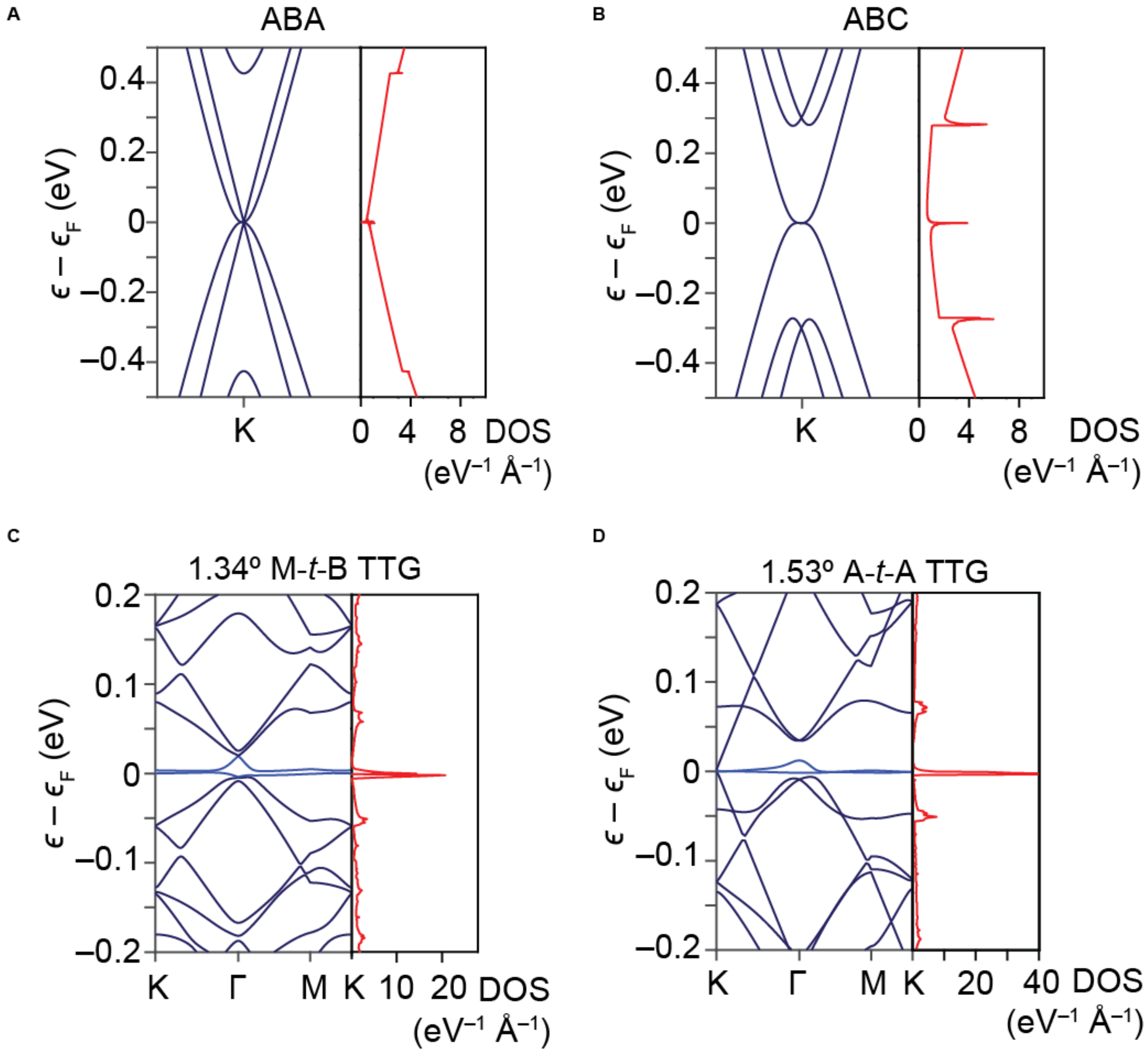

Supplementary Fig. 1: **Band structures and DOS. A–D,** Computed electronic band structures (see Methods) for ABA (**A**), ABC (**B**), 1.34° M-*t*-B (**C**), and 1.53° A-*t*-A (**D**).

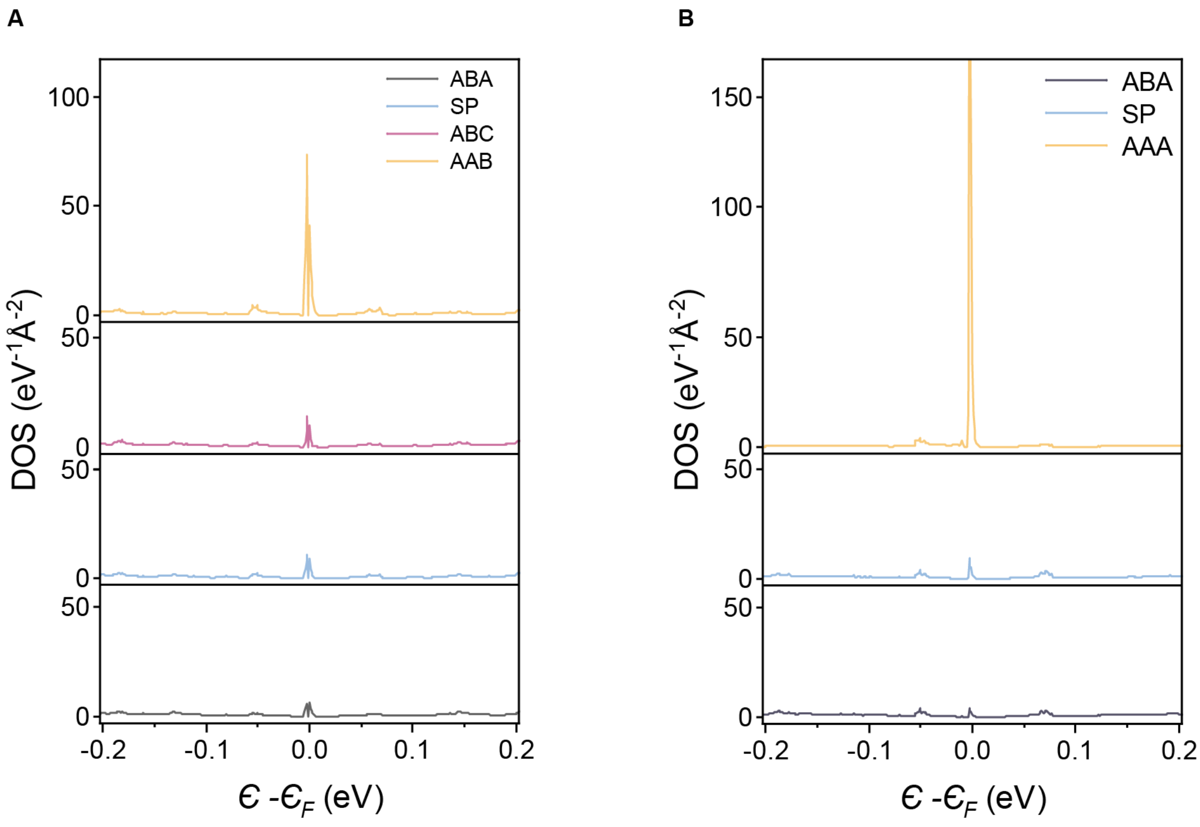

Supplementary Fig. 2: **Local density of states of twisted trilayer graphene.**
(**A**) Local density of states (LDOS) in 1.34° M-t-B. The yellow, red, blue, and black curves are the LDOS profile at AAB, ABC, saddle point (SP), and ABA regions respectively. (**B**) Local density of states (LDOS) in 1.53° M-t-B. The yellow, blue, and black curves are the LDOS profile at AAA, SP, and ABA regions respectively.

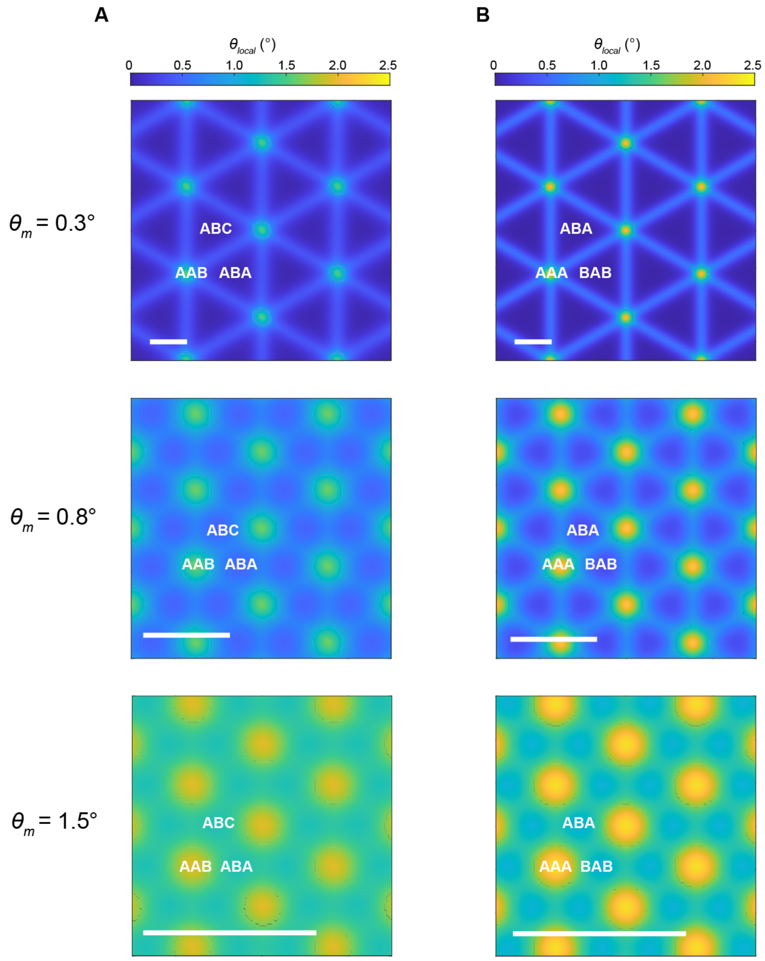

Supplementary Fig. 3: Local twist angle maps for relaxed M-*t*-B and A-*t*-A structures at different twist angles.
Calculated local rotation of M-*t*-B structures (in (**A**)) and A-*t*-A structures (in (**B**)) at 0.3°, 0.8°, and 1.5°. Scale bar: 10 nm.

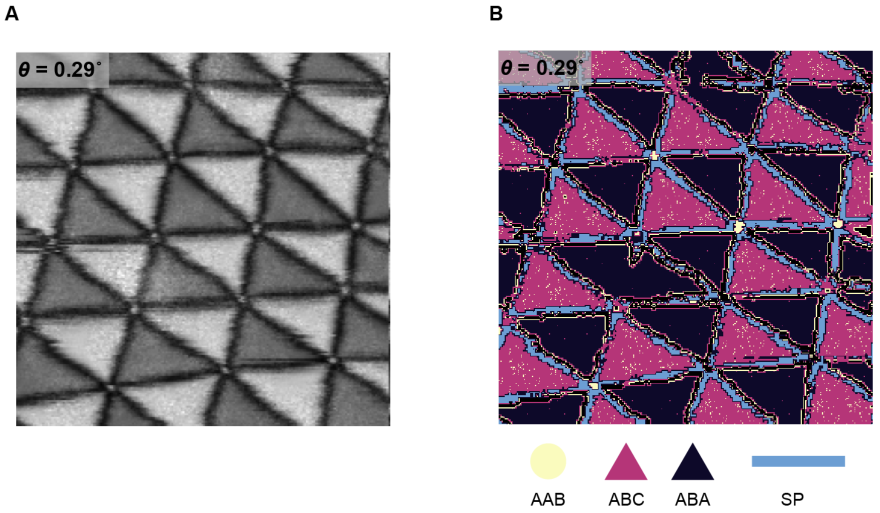

Supplementary Fig. 4: **Dark field TEM image of an M-*t*-B sample.**
(**A**) A representative dark field image that shows different stacking domains with distinctive contrast. (**B**) Fitted stacking domains based on different contrast.

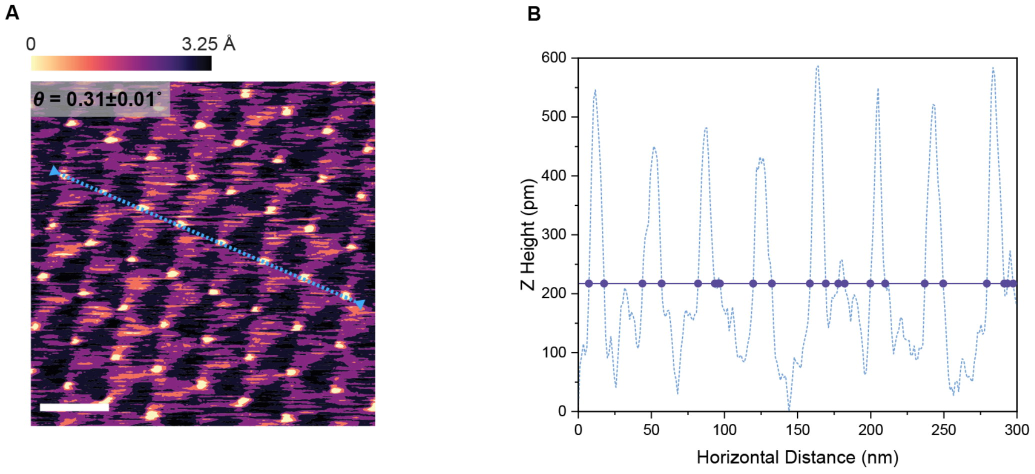

Supplementary Fig. 5: **STM area fraction analysis.**
(**A**) A representative STM image used for area fraction analysis of different stacking domains. The blue dash line was a line scan used for extracting AAB domain width. (**B**) Line scan Z height over a horizontal distance. The purple line represents the full-width-half-max cutoff used for estimating the AAB radius.

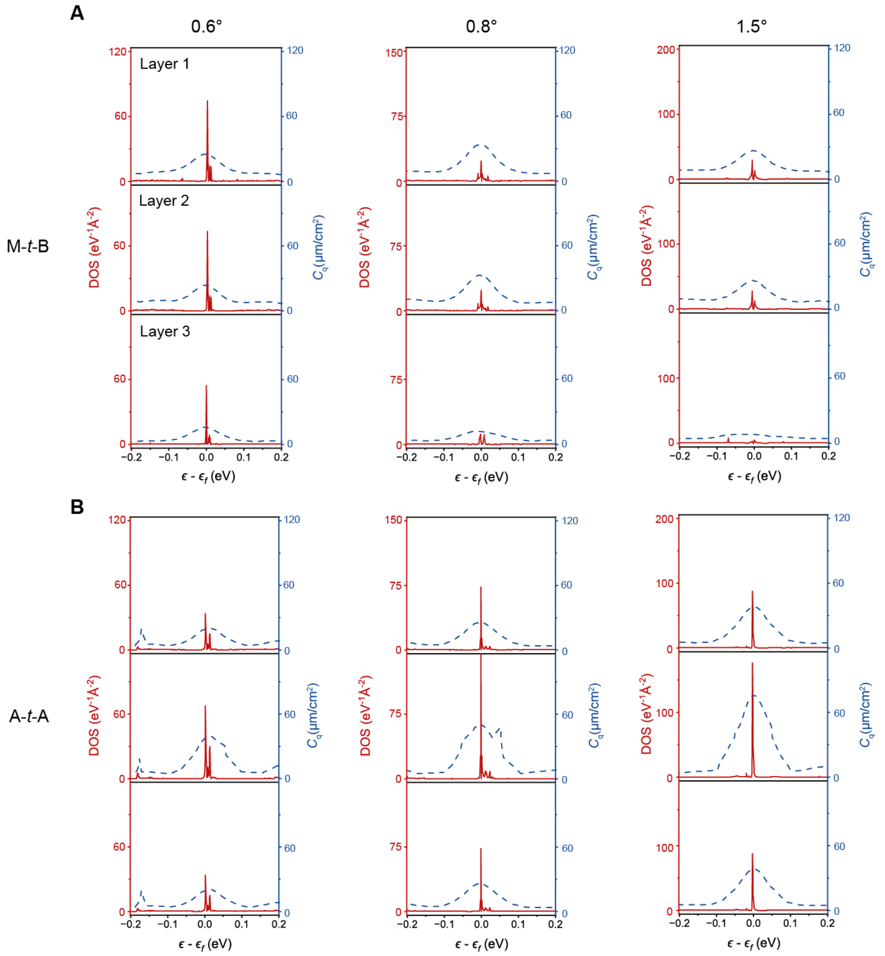

Supplementary Fig. 6: **Calculated layer-dependent local density of states of M-*t*-B and A-*t*-A on high energy stacking domains.**

Layer-dependent local density of states on AAB domains of different twist angles M-*t*-B in (**A**) and on AAA domains of different twist angles A-*t*-A in (**B**). The twist angles here refer to the local twist angles at these stacking domains without considering relaxation.

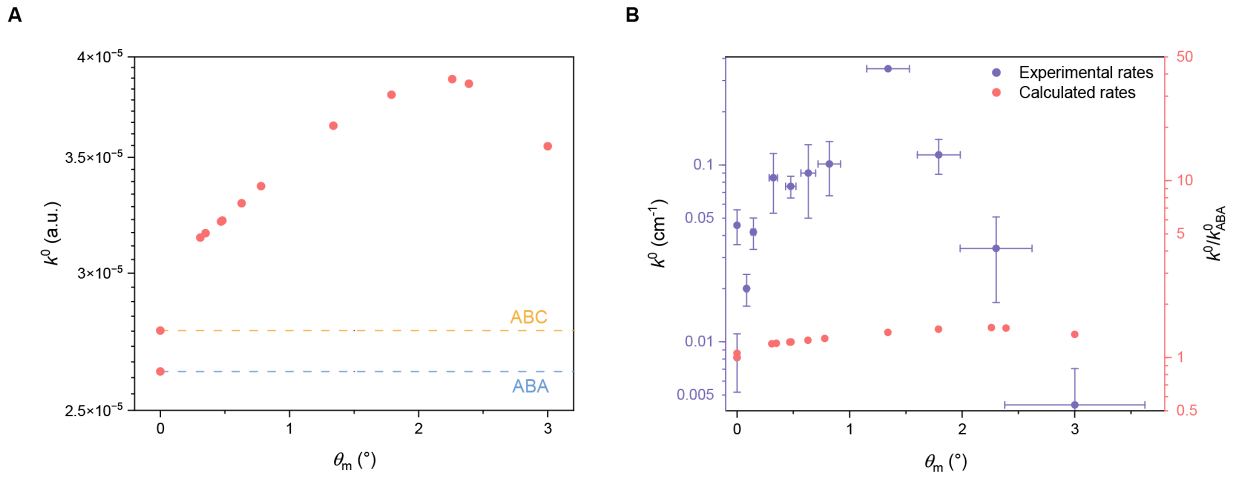

Supplementary Fig. 7: **Electron transfer rates calculated by Marcus-Hush-Chidsey theory.**
(**A**) Standard rates constant (k^0) versus twist angle θ_m calculated based on MHC theory. (**B**) Standard rate constants extracted from the experimental voltammograms as a function of twist angle (in blue) compared to calculated rates normalized by k^0_{ABA} (in red).

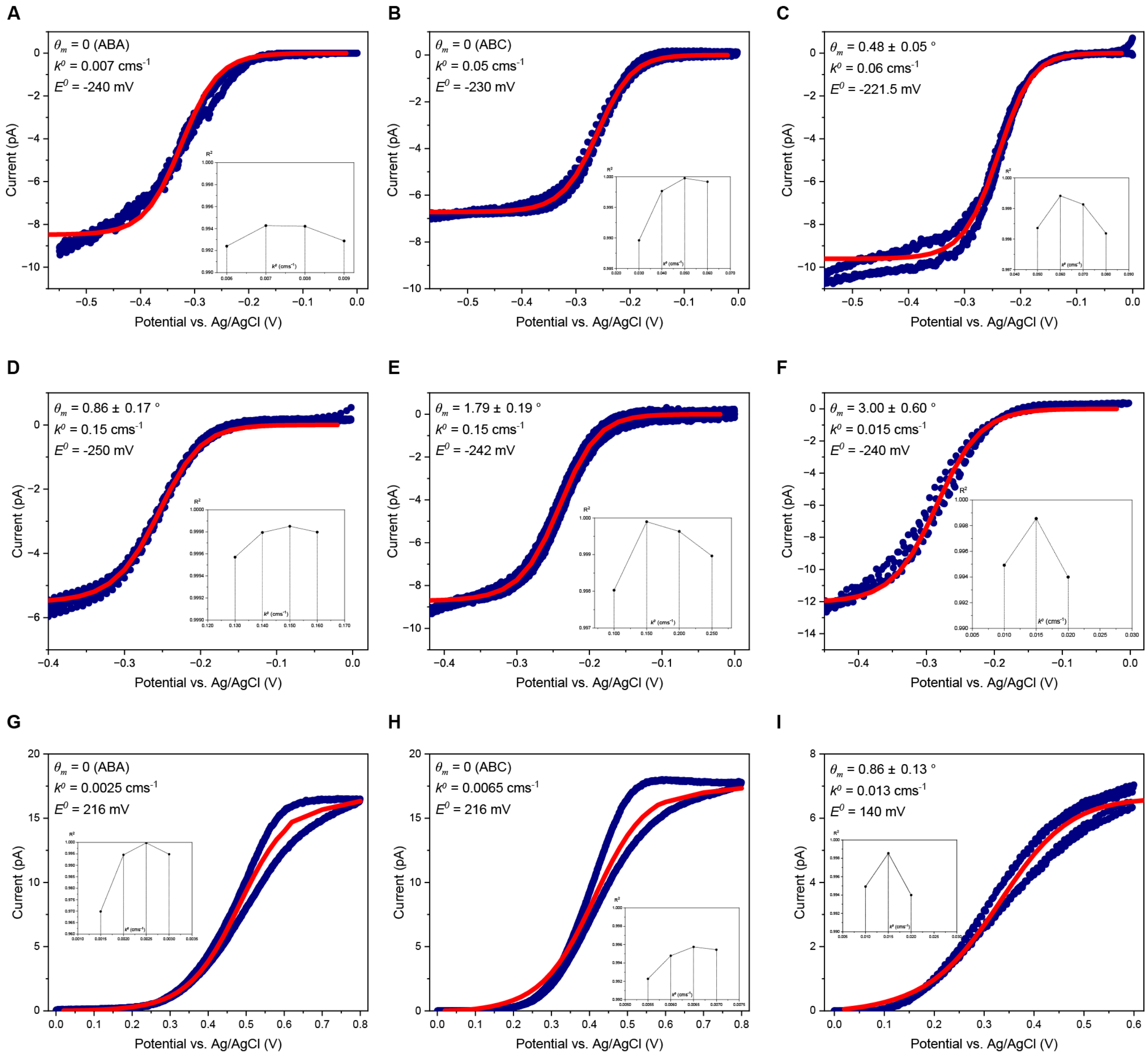

Supplementary Fig. 8: **Representative fittings for cyclic voltammograms.** Representative steady-state voltammograms (blue lines) of $Ru(NH_3)_6{}^{3+/2+}$ in (**A**)-(**F**) and $Co(phen)_3{}^{3+/2+}$ in (**G**)-(**I**) couple fit to simulations (red lines). Scan rate v = 100 mV/s. The red lines represent the simulated curve with the rate constant of best fit (or with maximum R^2).

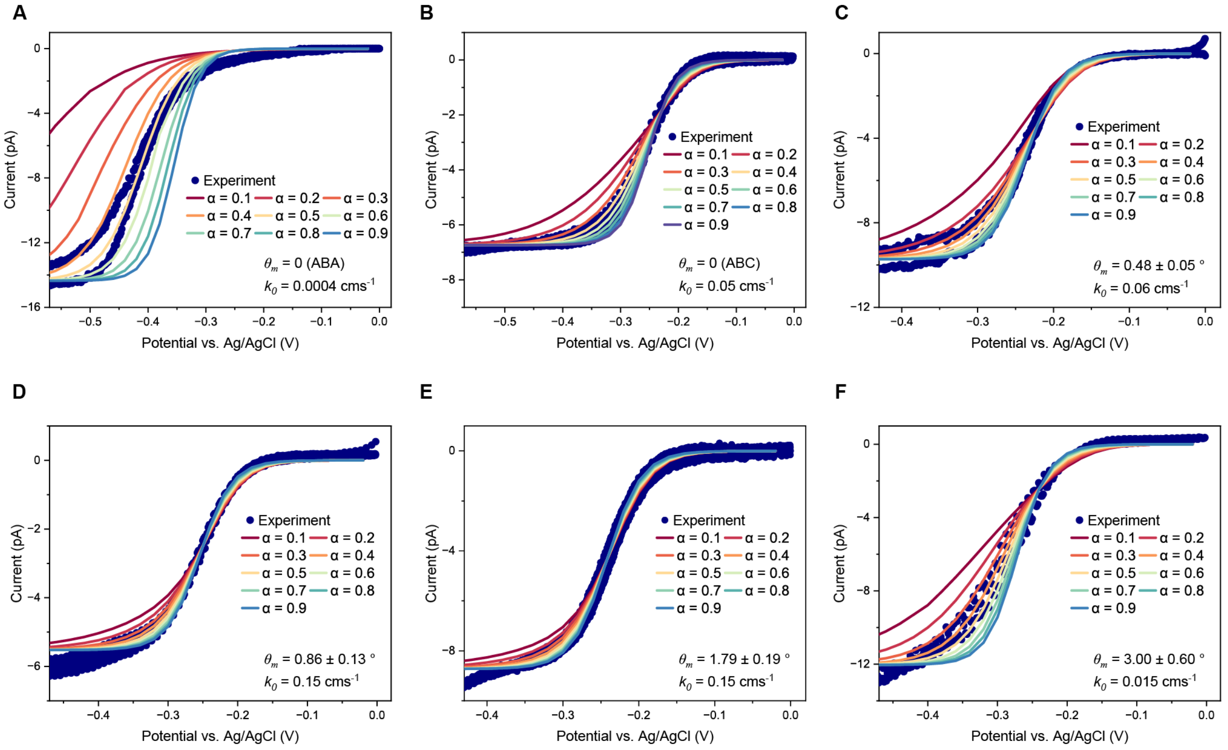

Supplementary Fig. 9: **Fitting of α in representative $Ru(NH_3)_6^{3+/2+}$ CVs.** Representative CVs of $Ru(NH_3)_6^{3+/2+}$ measured on various M-*t*-B samples of twist angles in blue dots. The colorful lines are simulated CVs in Comsol based on different transfer coefficients α. Across all twist angles, $\alpha = 0.5$ was the best fit.

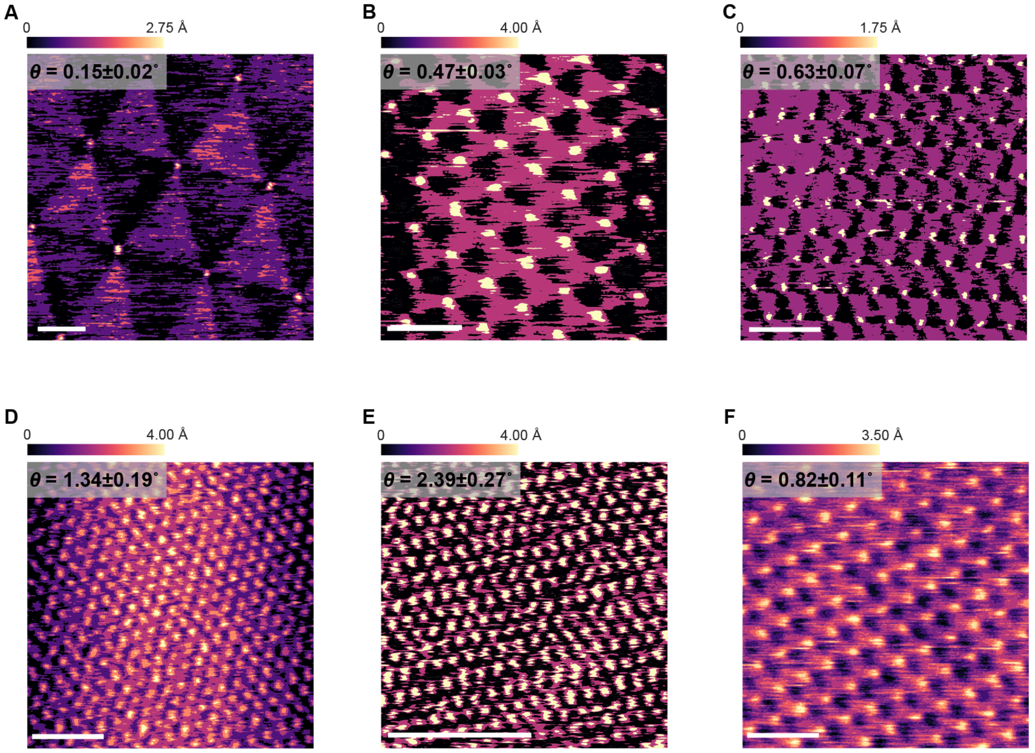

Supplementary Fig. 10: **Representative STM images of twisted trilayer samples.** Representative constant current STM images of various M-*t*-B in (**A**) - (**E**) and B-*t*-M in (**F**) of different twist angles.

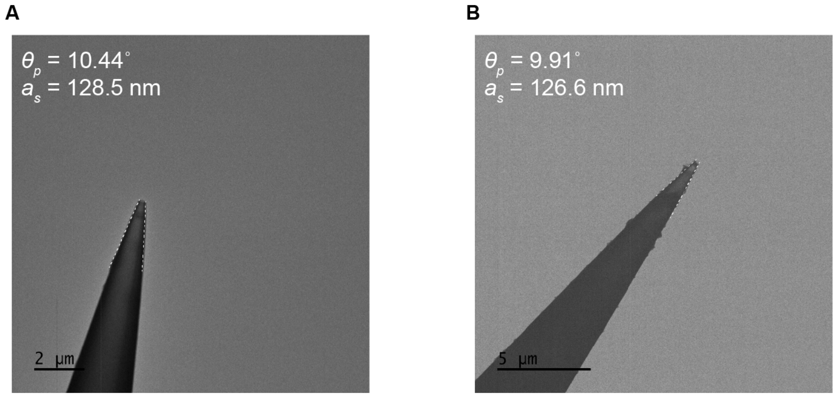

Supplementary Fig. 11: **TEM bright field images of quartz pipettes.**
Representative TEM images of quartz pipettes used in SECCM before, in (**A**), and after experiments in (**B**).

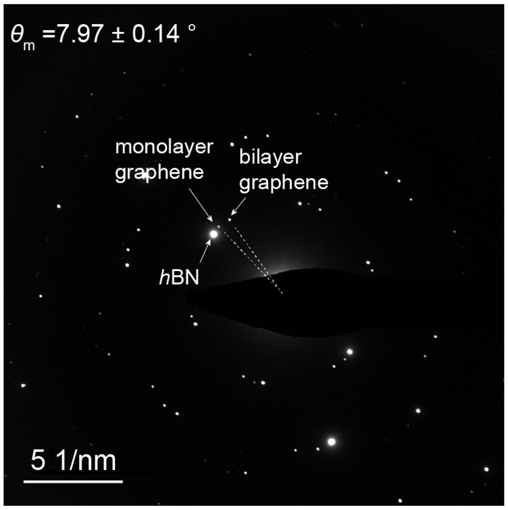

Supplementary Fig. 12: **Selected area diffraction pattern of a large twist angle M-*t*-B sample.**
The sample angle was determined by measuring the angle between the line extensions to the center of the bilayer and the monolayer peak.

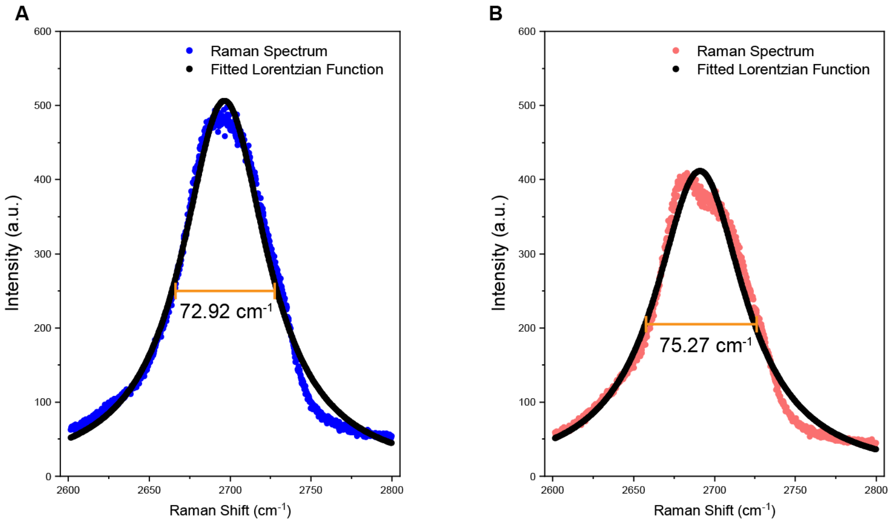

Supplementary Fig. 13: **Raman spectra of trilayer graphene**
(**A**) Representative Raman spectrum of ABA graphene from 2550 - 2800 cm^{-1} (in blue) and a Lorentzian fitting of the spectrum. The full-width-half-max was determined to be 72.92 cm^{-1}. (**B**) Representative Raman spectrum of ABC graphene from 2550 - 2800 cm^{-1} (in red) and a Lorentzian fitting of the spectrum. The full-width-half-max was determined to be 75.27 cm^{-1}.

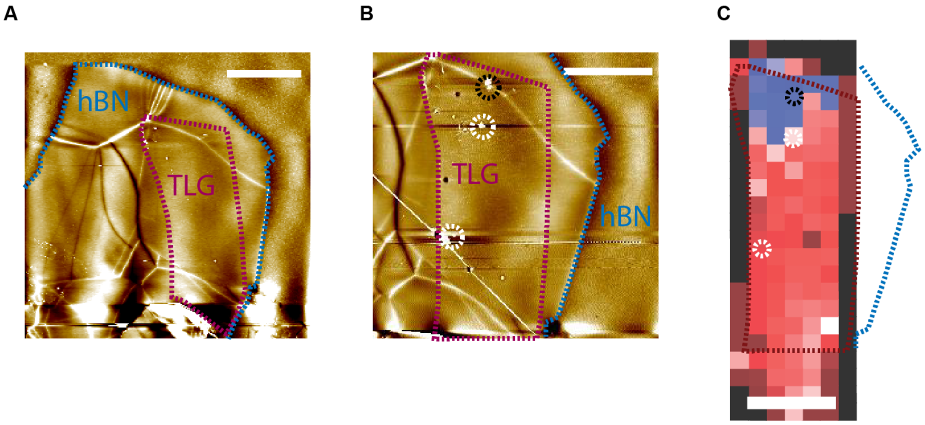

Supplementary Fig. 14: **Atomic force microscope (AFM) images of a natural trilayer graphene stack.**

AFM images measured on the same sample before in (**A**) and after (**B**) SECCM experiments. The red trace shows the trilayer graphene and the blue trace shows the bottom hBN. Black and white dotted circles highlighted residues from the measurements. Both images were taken on a Park AFM NX10 system with non-contact mode with a set point of 10 nm. (**C**) Raman map overlayed with the traces of the trilayer graphene and hBN. The measurements on the black dotted circle were used as rates on ABA graphene and those on the white circles were used as rates on ABC. Scale bar: 10 µm.

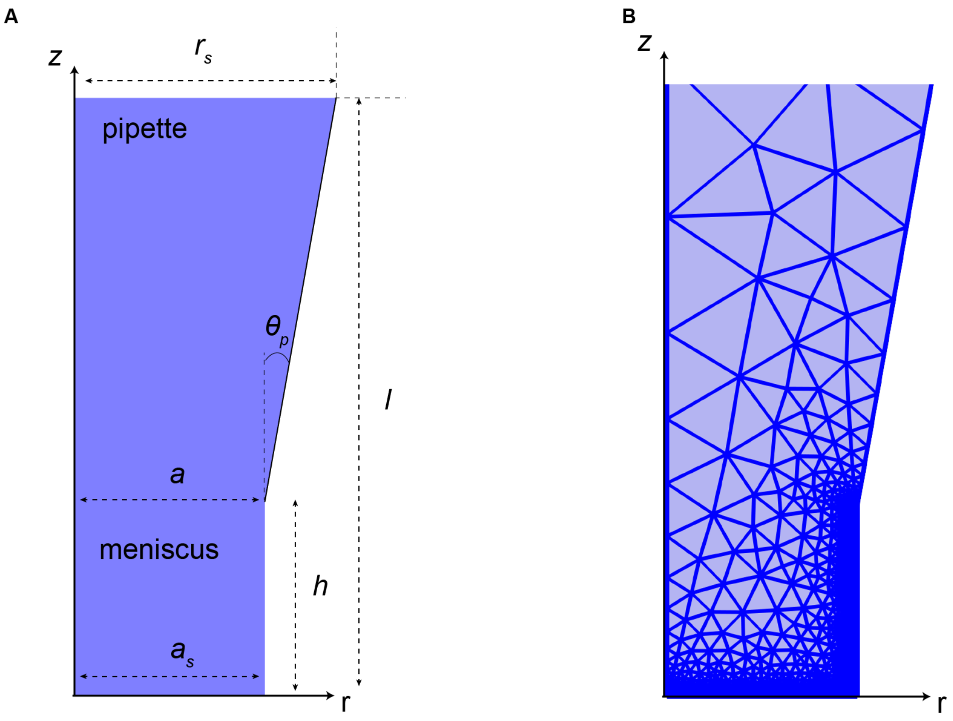

Supplementary Fig. 15: **Model geometry for the COMSOL simulation.**
(**A**) The model used to emulate the pipette and meniscus geometry. (**B**) A representative mesh used for the simulation.

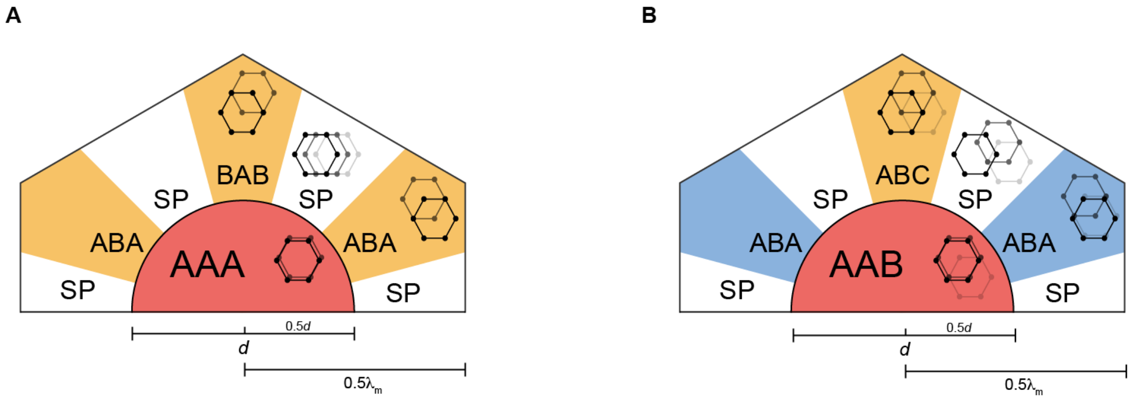

Supplementary Fig. 16: **Rigid moiré of A-*t*-A and M-*t*-B.**
The model unit cell used to calculate rigid moiré area fraction of A-*t*-A in (**A**) and M-*t*-B in (**B**). d is the diameter of the AAA or AAB domain. We assumed that d is half of λ_m which is the moiré wavelength.

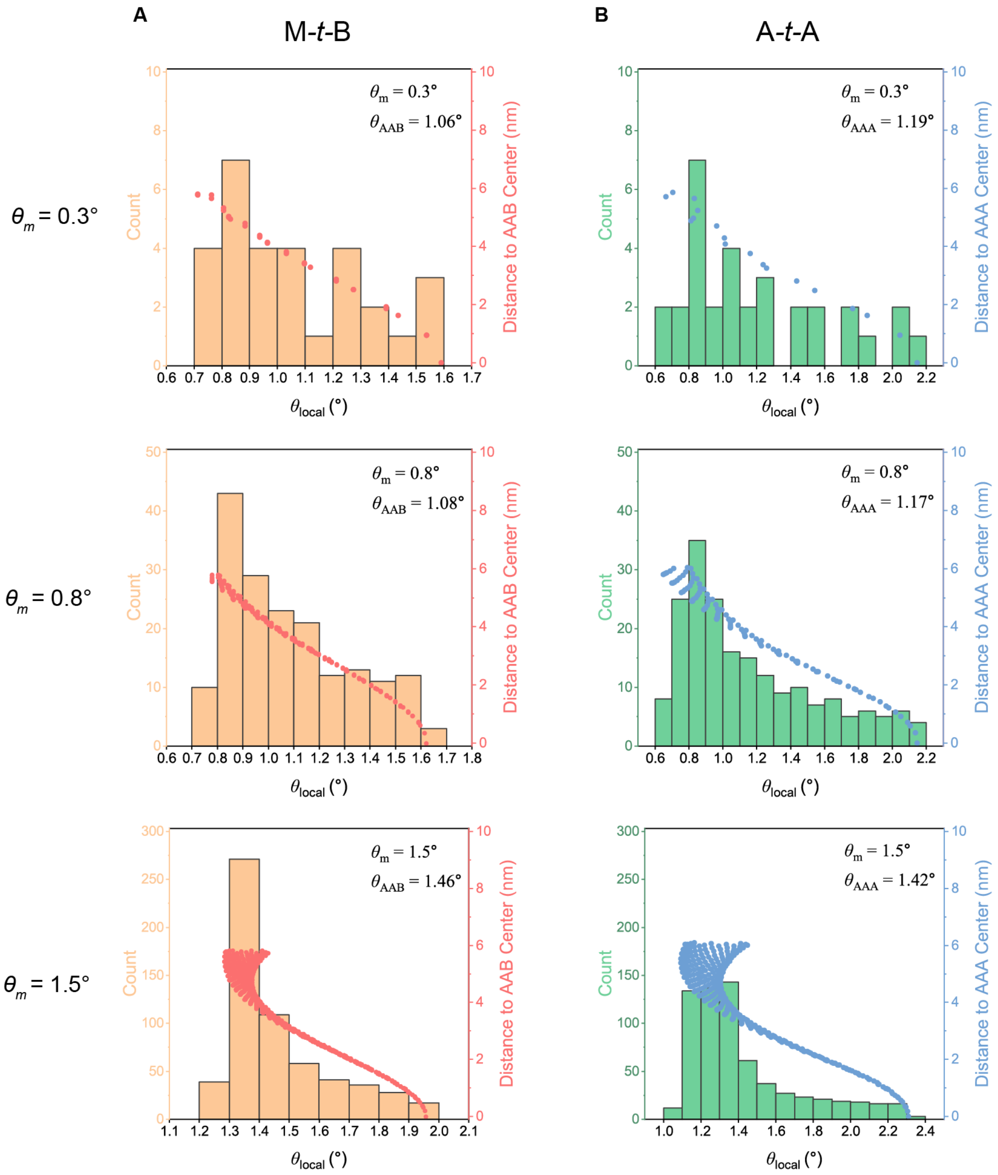

Supplementary Fig. 17: **The distribution of calculated local twist angle within AAB and AAA domains in relaxed moiré structures.**

Calculated local rotation distribution based on the distance from AAB centers in M-*t*-B structures (in (**A**)) and AAA centers in A-*t*-A structures (in (**B**)) at 0.3°, 0.8°, and 1.5°. The radii of the AAA/AAB stacking domains were calculated based on the STM measurements (see Supplementary Text).

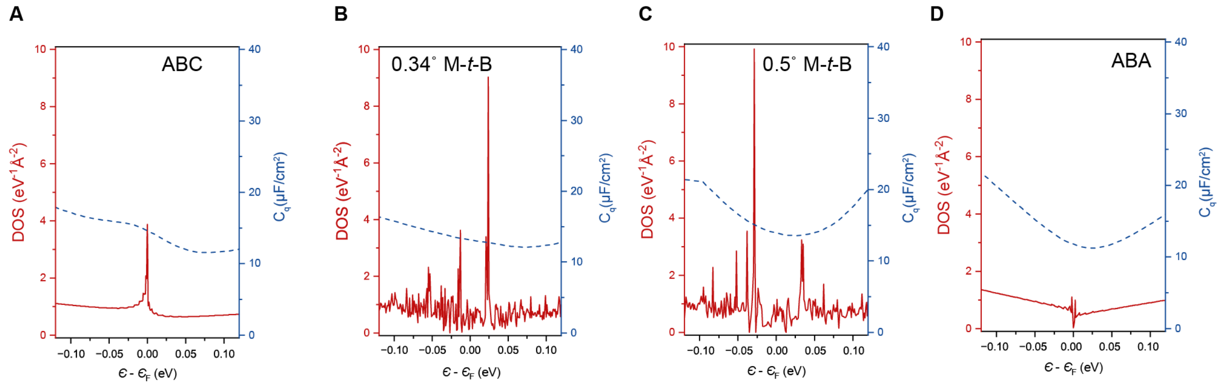

Supplementary Fig. 18: **LDOS and quantum capacitance of low angle M-*t*-B at the ABC domain compared with ABC and ABA graphene.**
The red curves are the calculated density of states at ABC (in (**A**)), ABA (in (**D**)), and local density at the ABC domain of 0.34° M-*t*-B (in (**B**)), 0.5° M-*t*-B (in (**C**)). The blue curves are the corresponding quantum capacitance with the LDOS and DOS assuming $T = 300$ K. At twist angles larger than $\sim 0.3°$ in M-*t*-B, the flat-band at the ABC sites deviates away from the Fermi level and the overall quantum capacitance approach that of the ABA graphene. This suggests the local domains depart from commensurate ABC stacking as the twist angle increases. No reconstruction effects have been considered in the calculation of LDOS. The noise LDOS of the twisted sample pertains to the finer k spacing used in those calculations.

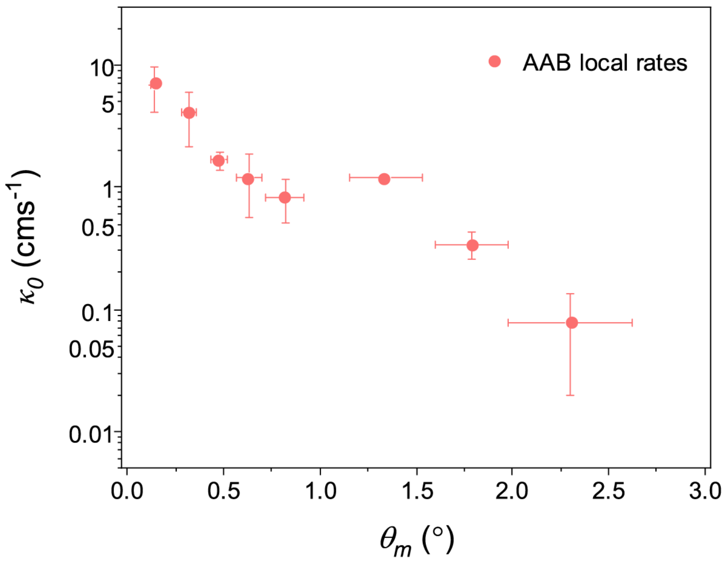

Supplementary Fig. 19: **Calculated AAB rates for samples of small twist angles.** Calculated local electron transfer rate κ_{AAB} of samples with twist angle smaller than 2.5 °. Each red dot denotes the average of κ^0_{AAB} on samples within a standard deviation of the mean twist angle. The horizontal and vertical error bars represent the standard deviations of θ_m and the standard error of κ^0_{AAB}. κ^0_{AAB} is consistent (within one order of magnitude) at small twist angles, but it generally decreases as the twist angle increases. This result hints at the possibility of the local ABC stacking domains deviating from commensurate states as the twist angle increases. Therefore, the local rates at the ABC domain were overestimated, and hence AAB rates were potentially underestimated at larger angles.

3 Supplementary Tables

Supplementary Table 1: Twist angles and rates of $Ru(NH_3)_6^{3+/2+}$ reduction on A-*t*-A, B-*t*-M and M-*t*-B samples. The rates of M-*t*-B were plotted in Fig. 2C. The rates of A-*t*-A, B-*t*-M and M-*t*-B samples at 0.82° were plotted in Fig.2C inset.

Sample name	Sample type	θ_m (°)	k^0 (cm/s)
ttl34	A-*t*-A	1.535 ± 0.163	0.112 ± 0.013
ttl40	A-*t*-A	0.819 ± 0.111	0.035 ± 0.003
ttl32	B-*t*-M	0.817 ± 0.127	0.023 ± 0.009
ttl6	M-*t*-B	0.633 ± 0.065	0.090 ± 0.040
ttl11	M-*t*-B	1.792 ± 0.190	0.114 ± 0.025
ttl17	M-*t*-B	1.342 ± 0.189	0.350 ± 0.000
ttl21	M-*t*-B	7.979 ± 0.140	0.002 ± 0.000
ttl24	M-*t*-B	3.001 ± 0.623	0.004 ± 0.003
ttl22&31	M-*t*-B	0.323 ± 0.037	0.085 ± 0.031
ttl20&30	M-*t*-B	0.477 ± 0.046	0.076 ± 0.011
ttl4&7&26	M-*t*-B	2.302 ± 0.320	0.034 ± 0.017
ttl13&15	M-*t*-B	0.085 ± 0.012	0.020 ± 0.004
ttl16&29	M-*t*-B	0.145 ± 0.019	0.042 ± 0.008
ttl3&41	M-*t*-B	0.821 ± 0.100	0.101 ± 0.034

Supplementary Table 2: Area fraction (AF) and local rates at the high energy stacking domains (AAA, BAA, and ABB respectively) of A-*t*-A, B-*t*-M and M-*t*-B samples and area fraction of saddle points (SP AF).

Sample name	Sample type	θ_m (°)	AF	SP AF	κ^0 (cm/s)
ttl6	M-t-B	0.633 ± 0.065	0.062	0.350	1.199 ± 0.911
ttl22&31	M-t-B	0.323 ± 0.037	0.016	0.239	4.110 ± 1.943
ttl20&30	M-t-B	0.477 ± 0.046	0.035	0.309	1.661 ± 0.301
ttl16&29	M-t-B	0.145 ± 0.019	0.003	0.123	6.814 ± 2.715
ttl3&41	M-t-B	0.821 ± 0.100	0.104	0.360	0.833 ± 0.329
ttl32	B-t-M	0.817 ± 0.127	0.104	0.360	0.165 ± 0.009
ttl34	A-t-A	1.535 ± 0.163	0.302	0.313	0.357 ± 0.044
ttl40	A-t-A	0.819 ± 0.111	0.114	0.360	0.260 ± 0.025

Supplementary Table 3: Area fraction of different stacking domains in a rigid moiré.

Sample type	Stacking domain	Area fraction
M-t-B	AAB	0.302
M-t-B	ABC	0.192
M-t-B	AAC	0.192
M-t-B	SP	0.313
A-t-A	AAA	0.302
A-t-A	ABA	0.192
A-t-A	BAB	0.192
A-t-A	SP	0.192

References

(1) Yu, Y.; Zhang, K.; Parks, H.; Babar, M.; Carr, S.; Craig, I. M.; Van Winkle, M.; Lyssenko, A.; Taniguchi, T.; Watanabe, K., et al. Tunable angle-dependent electrochemistry at twisted bilayer graphene with moiré flat bands. *Nature Chemistry* **2022**, *14*, 267–273.

(2) Kirkman, P. M.; Güell, A. G.; Cuharuc, A. S.; Unwin, P. R. Spatial and temporal control of the diazonium modification of sp2 carbon surfaces. *Journal of the American Chemical Society* **2014**, *136*, 36–39.

(3) Patel, A. N.; McKelvey, K.; Unwin, P. R. Nanoscale electrochemical patterning reveals the active sites for catechol oxidation at graphite surfaces. *Journal of the American Chemical Society* **2012**, *134*, 20246–20249.

(4) Wang, Y.; Limon-Petersen, J. G.; Compton, R. G. Measurement of the diffusion coefficients of $Ru(NH_3)_6^{3+}$ and $Ru(NH_3)_6^{2+}$ in aqueous solution using microelectrode double potential step chronoamperometry. *Journal of Electroanalytical Chemistry* **2011**, *652*, 13–17.

(5) Velicky, M.; Bradley, D. F.; Cooper, A. J.; Hill, E. W.; Kinloch, I. A.; Mishchenko, A.; Novoselov, K. S.; Patten, H. V.; Toth, P. S.; Valota, A. T., et al. Electron transfer kinetics on mono-and multilayer graphene. *ACS Nano* **2014**, *8*, 10089–10100.

(6) Güell, A. G.; Cuharuc, A. S.; Kim, Y.-R.; Zhang, G.; Tan, S.-y.; Ebejer, N.; Unwin, P. R. Redox-dependent spatially resolved electrochemistry at graphene and graphite step edges. *ACS Nano* **2015**, *9*, 3558–3571.

(7) Kazmierczak, N. P.; Van Winkle, M.; Ophus, C.; Bustillo, K. C.; Carr, S.; Brown, H. G.; Ciston, J.; Taniguchi, T.; Watanabe, K.; Bediako, D. K. Strain fields in twisted bilayer graphene. *Nature Materials* **2021**, *20*, 956–963.

(8) Carr, S.; Massatt, D.; Torrisi, S. B.; Cazeaux, P.; Luskin, M.; Kaxiras, E. Relaxation and domain formation in incommensurate two-dimensional heterostructures. *Phys. Rev. B* **2018**, *98*, 224102.

(9) Zhou, S.; Han, J.; Dai, S.; Sun, J.; Srolovitz, D. J. van der Waals bilayer energetics: Generalized stacking-fault energy of graphene, boron nitride, and graphene/boron nitride bilayers. *Phys. Rev. B* **2015**, *92*, 155438.

(10) Cazeaux, P.; Luskin, M.; Massatt, D. Energy minimization of two dimensional incommensurate heterostructures. *Archive for Rational Mechanics and Analysis* **2020**, *235*, 1289–1325.

(11) Kaxiras, E.; Duesbery, M. S. Free energies of generalized stacking faults in Si and implications for the brittle-ductile transition. *Physical Rev. Letters* **1993**, *70*, 3752.

(12) Zhu, Z.; Carr, S.; Massatt, D.; Luskin, M.; Kaxiras, E. Twisted trilayer graphene: A precisely tunable platform for correlated electrons. *Phys. Rev. Lett.* **2020**, *125*, 116404.

(13) Kresse, G.; Hafner, J. Ab initio molecular dynamics for liquid metals. *Phys. Rev. B* **1993**, *47*, 558.

(14) Kresse, G.; Furthmüller, J. Efficiency of ab-initio total energy calculations for metals and semiconductors using a plane-wave basis set. *Computational materials science* **1996**, *6*, 15–50.

(15) Kresse, G.; Furthmüller, J. Efficient iterative schemes for ab initio total-energy calculations using a plane-wave basis set. *Phys. Rev. B* **1996**, *54*, 11169.

(16) Peng, H.; Yang, Z.-H.; Perdew, J. P.; Sun, J. Versatile van der Waals density functional based on a meta-generalized gradient approximation. *Physical Rev. X* **2016**, *6*, 041005.
